# Transcriptome analysis of sweet potato responses to potassium deficiency

**DOI:** 10.1186/s12864-022-08870-5

**Published:** 2022-09-15

**Authors:** Fang Wang, Wen-Fang Tan, Wei Song, Song-Tao Yang, Shuai Qiao

**Affiliations:** Environment-friendly Crop Germplasm Innovation and Genetic Improvement Key Laboratory of Sichuan Province, Crop Research Institute, Sichuan Academy of Agricultural Sciences, Chengdu, 610066 Sichuan China

**Keywords:** Sweet potato, Low-K^+^ stress, Transcriptomic, DEGs

## Abstract

**Background:**

As one of three essential nutrients, potassium is regarded as a main limiting factor for growth and development in plant. Sweet potato (*Ipomoea batatas* L.) is one of seven major food crops grown worldwide, and is both a nutrient-rich food and a bioenergy crop. It is a typical ‘K-favoring’ crop, and the level of potassium ion (K^+^) supplementation directly influences its production. However, little is known about the transcriptional changes in sweet potato genes under low-K^+^ conditions. Here, we analyzed the transcriptomic profiles of sweet potato roots in response to K^+^ deficiency to determine the effect of low-K^+^ stress on this economically important crop.

**Results:**

The roots of sweet potato seedlings with or without K^+^ treatment were harvested and used for transcriptome analyses. The results showed 559 differently expressed genes (DEGs) in low and high K^+^ groups. Among the DEGs, 336 were upregulated and 223 were downregulated. These DEGs were involved in transcriptional regulation, calcium binding, redox-signaling, biosynthesis, transport, and metabolic process. Further analysis revealed previously unknow genes involved in low-K^+^ stress, which could be investigated further to improve low K^+^ tolerance in plants. Confirmation of RNA-sequencing results using qRT-PCR displayed a high level of consistency between the two experiments. Analysis showed that many auxin-, ethylene- and jasmonic acid-related genes respond to K^+^ deficiency, suggesting that these hormones have important roles in K^+^ nutrient signaling in sweet potato.

**Conclusions:**

According to the transcriptome data of sweet potato, various DEGs showed transcriptional changes in response to low-K^+^ stress. However, the expression level of some kinases, transporters, transcription factors (TFs), hormone-related genes, and plant defense-related genes changed significantly, suggesting that they have important roles during K^+^ deficiency. Thus, this study identifies potential genes for genetic improvement of responses to low-K^+^ stress and provides valuable insight into the molecular mechanisms regulating low K^+^ tolerance in sweet potato. Further research is required to clarify the function of these DEGs under low-K^+^ stress.

**Supplementary Information:**

The online version contains supplementary material available at 10.1186/s12864-022-08870-5.

## Background

Potassium ions (K^+^) are an essential macronutrients for plant growth and development. Unlike nitrate (NO_3_^−^) and phosphate (H_2_PO_4_^−^), it can assimilate into organic matter. K^+^ is the most abundant cation in higher plants, in which it participates in many fundamental processes, including enzyme activation, membrane potential, membrane transport, and turgor maintenance, as well as determining the yield and quality of crops [1–3]. As one of the most important cations, it comprises 2–10% of the plant dry weight and its concentration in fresh tissues is in the range 10–100 mM [4, 5].

To improve their K utilization efficiency (KUE), plants have evolved multiple complex uptake mechanisms that operate at low external and high environmental K^+^ concentrations [6–8]. K^+^ is absorbed from the soil into plant cells through K^+^ transport components, such as K^+^ transporters and channels [9, 10]. AKT1 from the plant Shaker family was the first K^+^ channel identified and functions at a wide range of external K^+^ concentrations (approximately 0.01–10 mM); it is considered the main channel mediating K^+^ influx into root cells [11–13]. HAK5 from the HAK/KUP/KT (high-affinity K^+^/K^+^ uptake/K^+^ transporter) family is a high-affinity K^+^ transporter that contributes approximately half the K^+^ absorption under K^+^-deficient conditions in Arabidopsis [14, 15]. AKT1 and HAK5 are the most important transport proteins mediating almost all K^+^ absorption in Arabidopsis [2, 16, 17].

K^+^ is involved in multiple abiotic and biotic stresses signal pathways in plants [18–20]. As potassium transporters, some HAK/KUP/KT family genes were reported to respond to drought and salt stresses [21, 22]. For example, the HAK/KUP/KT family K^+^ transport ZmHAK4 has distinct roles in promoting shoot Na^+^ exclusion and salt tolerance [23]. OsHAK1, a high-affinity K^+^ transporter, positively regulates response to drought stress in rice [24]. KUP9 is a member of the HAK/KUP/KT family, and controls primary root growth in Arabidopsis [25]. In addition, K^+^ has important physiological roles in cell division, as well as sugar translocation to fruits via the phloem, fruit coloration, shelf life, and the shipping quality of many horticultural crops [26–28]. Low K^+^ reduces photosynthesis, impairs the supply of photosynthates to sink organs, and promotes sucrose export to phloem from source leaves. This affects not only yield formation, but also quality parameters in, for example, wheat, potato, and grape [29]. In sweet potato (*Ipomoea batatas* L.), it was reported that K^+^ application promoted starch accumulation and storage root yield through regulating the activity and transcription of genes involved in sucrose-to-starch conversion [30]. Several studies have shown various effects of different forms of K on fruit yield; for example, the increasing yield of K-treated trees was correlated with an increase in fruit weight [28, 31]. K^+^ is the major osmotic solute imported into fibers and elongating fiber cells require abundant K to maintain cell turgor pressure, which they acquire via K^+^ transporter genes that are preferentially expressed in elongating fibers [32, 33]. K^+^ deficiency reduced cotton fiber strength and fiber length, and also accelerated cotton senescence and reduce production [34–36]. K-deficient plants are also easily infected by diseases and pests [37–39].

Sweet potato (*Ipomoea batatas* L.; Convolvulaceae) is a tuber crop and one of the most important crops in China and many other countries [40, 41]. It has 2n = 6x = 90 chromosomes and the haplotype-resolved genome sequence of sweet potato enables the investigation of gene functions [42]. However, the genome of sweet potato is highly heterozygous and generally self-incompatible, which poses numerous challenges for conventional breeding [40]. Sweet potato is a typical ‘K-favoring’ crop, and has high nutritive value. It is a cheap source of energy, carbohydrates, vitamins, K, iron, fiber, and protein [43]; however, little progress has been made in understanding changes in the transcription of sweet potato genes under low-K^+^ stress. Given that, of the three major nutritional elements (N, P and K), K is the nutrient in highest demand, research is required into the effect of its deficiency on sweet potato.

RNA sequencing (RNA-seq) technologies have become an important tool to identify differentially expressed genes (DEGs) at the transcriptome level and provide valuable information to accelerate studies in plants, especially for crops with complex genomes [44]. These technologies can be used to help understand all aspects of K^+^ management in plants, including processes related to growth, development, metabolism, and stress resistance [45]. In the present study, second sequencing technology was used to monitor the transcriptome profiles of sweet potato roots in response to K^+^ deficiency and to establish a useful database for transcriptome sequencing and DEGs under low-K^+^ stress. These results will further our understanding of the molecular mechanisms involved in, and provide novel insights into, the response of sweet potato to low-K^+^ stress, as well as the potential genes involved.

## Results

### Effect of low-K^+^ stress on the phenotype, K^+^ content, and relative expression of marker genes in sweet potato seedlings

**Fig. 1:**
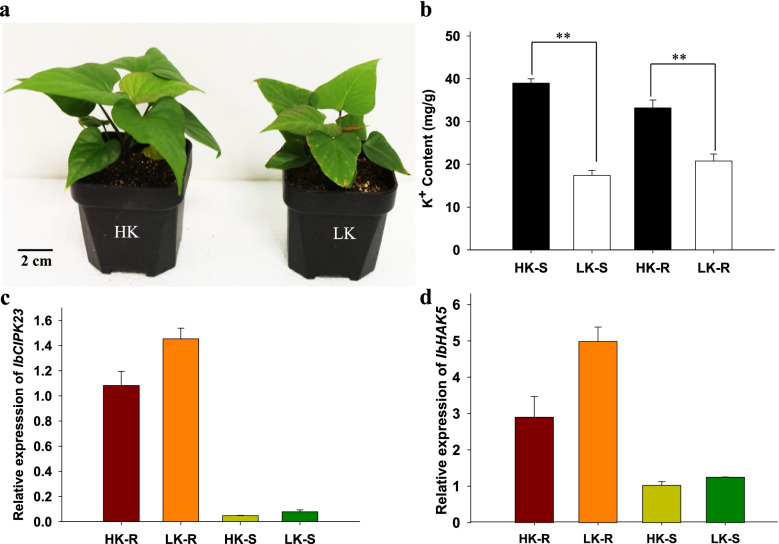
K^+^ deficiency phenotype and relation expression of low potassium maker gene. **a** Phenotype comparison between HK (Sufficient potassium) and LK (Low potassium) of sweet potato seedling. Sweet potato stems were cut into 5 cm fragments and growth in plot full with vermiculite to two leaf stage. Seedlings were then treated with HK (1 mM K) and LK (0 mM K) Hoagland solution for 14 days, Bar = 2 cm. **b** K^+^ content measurement. Student’s *t* test (**P < 0.01) was used to analyze statistical significance, *n* = 4. **c** and **d** Relative expression of maker gene *IbHAK5* and *IbCIPK23* response to low-K^+^ stress, *n* = 4, HK-S: sufficient potassium shoot, HK-R: sufficient potassium root, LK-S: low potassium shoot, LK-R: low potassium root

To identify the phenotype of sweet potato seedlings under K^+^-deficient conditions, low-K^+^ stress (LK, 0 mM K^+^) was applied at the two-leaf seedling stage. After 14 days, the K^+^-deficient phenotypes were evaluated. Compared with control seedlings (HK, 1 mM K^+^), K^+^-deficient seedlings showed smaller shoots and chlorosis (Fig. 1a) and the K^+^ content was significantly reduced (Fig. 1b). The phenotype of sweet potato roots under low-K^+^ stress have been shown in Fig. S1a. The roots of sweet potato seedlings showed no obviously phenotype under normal and low-K^+^ stress and it may need more time to show obvious difference. These observed phenotypes were consistent with biomass and chlorophyll content measurements (Fig. S1b, c and e). In addition, these potassium deficiency phenotypes were consistent with those reported for other crops [45, 46]. In order to identify the potassium deficiency phenotype, the relative expression of the low-K^+^ stress marker genes was determined to further support the phenotype. CBL-interacting protein kinase 23 (*IbCIPK23*) and high-affinity K^+^ transporter 5 (*IbHAK5*) are two important genes involved in the response to low-K^+^ stress, and have crucial roles particularly under K^+^-deficient conditions [15, 47]. Thus genes were selected as controls for qPCR. The results showed that both genes were significantly upregulated in K^+^ deficient sweet potato roots (Fig. 1c and d). Phenotype and K^+^ content analyses showed that the sweet potato seedlings suffered low-K^+^ stress (Fig. 1a and b). Therefore, these materials were shown to be valid for obtaining transcriptome profiles of sweet potato under K^+^-deficient conditions.

### Transcriptome profiling in response to K^+^ deficiency

To assess gene expression in sweet potato roots under K^+^-deficient condition, a high-throughput transcriptome sequence analysis was conducted without a genome [44]. To ensure the reliability of the transcriptome data, three biological replicates were analyzed for each treatment. The roots of seedlings of ‘Taizhong6’, which is the variety cultivated in China, were treated under sufficient K^+^ and low-K^+^ stress for 14 days and RNA was extracted to obtain transcriptomic profiles. The quality of the sequencing data was sufficient to support further transcriptome analysis by paired-end sequencing using an Illumina NovaSeq6000 sequencing platform. Clean reads were obtained by removing adaptor sequences, unknown sequences, and low-quality reads from raw data. At the same time, Q20, Q30, GC-content, and the sequence duplication level of the clean data were calculated (Table S1). All analyses were based on high-quality clean reads. After quality control, ~ 22,350,759 and ~ 26,153,929 clean reads were obtained under low K^+^ and high K^+^ conditions, respectively (Table S1).

Trinity-v2.4.0 software was used to assemble clean reads and obtain unigenes. After analyzing the open-reading frame (ORF) findings, single-nucleotide polymorphisms (SNPs), and simple sequence repeats (SSRs), the genome was mapped for annotation and expression analysis. The quality of the assembly is closely related to the length and number of unigenes. In this study, the unique gene length showed wide range and those longer than 3000 bp accounted for 3.06% of genes (Fig. S2a). Unigenes were annotated in multiple databases, including NR database (the NCBI non-redundant protein sequences database); Swissprot (a manually annotated and non-redundant protein sequence database); Kyoto Encyclopedia of Genes and Genomes (KEGG); and Clusters of Orthologous Groups of proteins (KOG). In total, 91,690 unigenes were obtained (Table S2) and 38,516 unigenes were annotated (Table S3); all unigene sequences, annotation and expression data were collected in Additional files 2 and 5. The Venn diagram showing the annotated genes for each database is detailed in Fig. S2b.

**Fig. 2:**
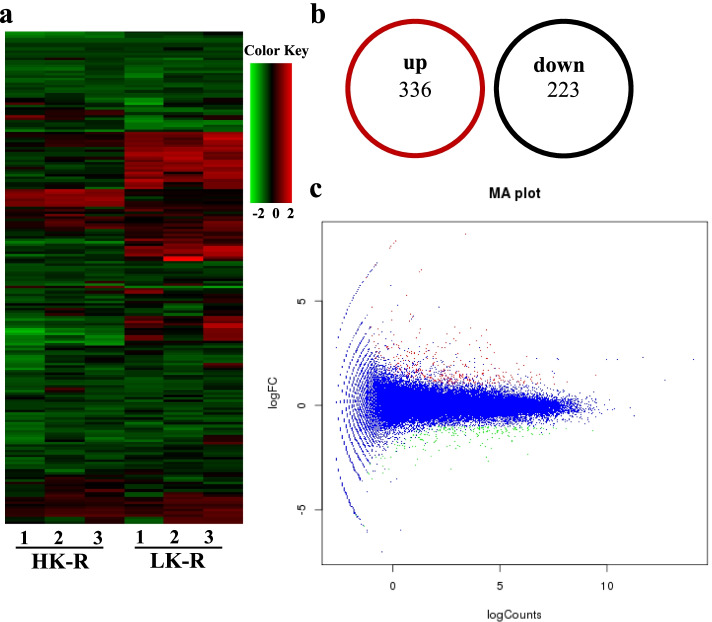
The heat map of DEGs and the number under low K^+^ stress. **a** Hierarchical clustering of all differentially expressed genes in the root of sweet potato seedlings, three biological parallels were used in each group. **b** The numbers of up-regulation and down-regulation genes of DEGs under K^**+**^ deficiency condition. **c** MA plot of the differently expressed genes in HK and LK. The x-axis shows the counts of differently expressed genes, and the y-axis shows fold change value of gene expression in LK. Blue dots indicate genes without significant differential expression. The red dots show significantly up-regulated DEGs, and the green dots show significantly down-regulated DEGs. HK: sufficient K; LK: Low-K^**+**^ treat

To investigate differences among the transcriptome sequence data, the hierarchical cluster analysis of different expression genes during K^+^ sufficient and K^+^ deficient conditions were conducted (Fig. 2a), indicating that there were obvious difference between HK (High K^+^) and LK (Low K^+^) condition. Statistical results showed that approximately 336 genes were observably upregulated and approximately 223 were downregulated in the low-K^+^ treatment (Fig. 2b). The numbers and fold changes of different expression genes under low K^+^ were clearly displayed in the MA plot, and the red and green dots indicate differently expressed genes (Fig. 2c).

### Transcriptome sequencing identified DEGs in sweet potato seedlings under low-K^+^ stress

In this study, 559 DEGs were observed under low-K^+^ stress, some of which were analyzed further (Table 1). These genes were selected based on their *p*-value and function, including transporters, TFs, cell wall-related genes, disease resistance-related genes, kinases, hormones, E3 ubiquitin ligases and uclacyanin (Table 1). Most of these genes are closely involved in low-K^+^ stress. For example, K^+^ and NO_3_^−^ are reported to be cooperatively involved in K^+^ uptake [48]. In the current study, high-affinity nitrate transporter 2.4 was upregulated, which implys this nitrate transporter may be involved in K^+^ homeostasis.

**Table 1:** List of DEGs from sweet potato roots (LK vs HK) identified in this study

Function	Annotation	Gene ID	Log_2_FC	*P*-value	Up or down regulate
Transporter	Sugar transporter sweet1	g39263.t1	−1.60	2.68E-04	down
	Sulfate transporter 3.4	g51451.t1	−1.07	1.90E-07	down
	High affinity nitrate transporter 2.4	g4703.t1	1.38	5.62E-05	up
	Auxin transporter (PIN 8)	g30544.t1	−1.65	1.40E-14	down
	Mitochondrial Fe^2+^ transporter MMT1	g69.t1	−1.28	1.78E-05	down
Transcription factor	Transcription factor MYC2	g33888.t1	3.18	1.88E-12	up
	Transcription factor TCP4	g63932.t1	−1.18	6.49E-08	down
	WRKY transcription factor 70	g20452.t1	1.12	1.42E-07	up
	Ethylene-responsive transcription factor 4	g5338.t1	2.26	2.84E-06	up
	NAC domain-containing protein 29	g42790.t1	1.39	1.60E-08	up
	MYB48	g13997.t1	1.67	1.90E-07	up
	Light-inducible transcription factor CPRF2	g12270.t1	−1.08	1.10E-04	down
Cell wall related	Wall-associated receptor kinase-like	g38476.t1	2.23	4.24E-04	up
	Lignin-forming anionic peroxidase	g8484.t1	1.38	1.52E-04	up
	Expansin-A2-like	g3926.t1	−2.04	8.11E-18	down
plant defense	Defensin J1–2	g12661.t1	1.42	2.92E-05	up
	Disease resistance response protein 206	g19608.t1	−1.06	1.06E-04	down
	Pathogenesis-related protein PR-1-like	g43100.t1	−1.15	2.51E-04	down
	Pathogenesis-related protein P2	g56218.t1	−1.08	2.57E-04	down
Kinase	calcium/calmodulin-dependent protein kinase type	g13127.t1	−1.28	8.95E-08	down
	Probable LRR receptor-like serine/threonine-protein kinase	g3822.t1	−1.24	9.88E-05	down
Hormone	JAZ7	g29758.t1	3.45	1.04E-12	up
	Ethylene receptor 2	g13882.t1	1.06	5.30E-4	up
	Auxin-induced protein	g3636.t1	−1.74	1.83E-16	down
E3 ubiquitin ligase	E3 ubiquitin ligase	g60180.t1	2.76	7.13E-06	up
Uclacyanin	Uclacyanin-2	g60212.t1	8.18	3.92E-59	up

**Fig. 3:**
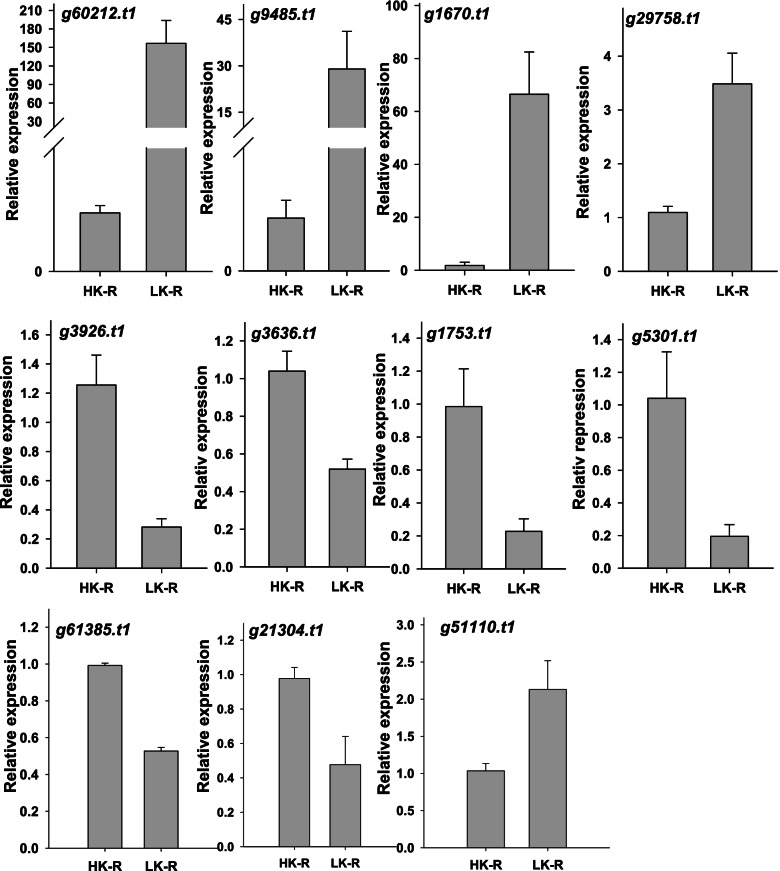
Confirmation the selected genes from transcriptomic profiles by real-time PCR. The sweet potato seedlings roots conducted with HK and LK for 14 d and used for qPCR verification. The experiments were repeated three times. The error bars represent ± SE. *n* = 3, HK: sufficient K; LK: Low-K^**+**^ treat; R: Root

To validate the quality of the gene activity profiles, 11 high DEGs were selected according to their *p*-values to compare their fragments per kilobase of exon per million fragments mapped (FPKM) reads and qPCR data (Table 2). Real-time PCR analyses further confirmed that the expression of the selected genes and the trends in gene expression changes determined by the two different approaches were largely consistent (Fig. 3). Thus, the DEGs determined in this study can be considered highly accurate.

**Table 2:** Selected top differently expressed gene in root under low and sufficient potassium condition

Gene ID	LK-1-FPKM	LK-2-FPKM	LK-3-FPKM	HK-1-FPKM	HK-2-FPKM	HK-3-FPKM	LgFC	*P*-value	Annotation
g60212.t1	38.392	53.082	29.505	0.322	0.171	0	8.1751	3.24E-54	Uclacyanin-2
g9485.t1	41.699	56.606	30.979	4.215	2.382	3.903	3.69659	7.73E-26	Glutathione S-transferase
g1670.t1	12.018	17.568	12.646	2.488	1.622	3.896	2.78176	6.52E-12	MADS-box transcription factor
g51110.t1	47.017	64.208	46.117	13.764	16.146	17.689	1.79262	2.01E-16	PEBP-like protein
g3926.t1	15.152	14.347	17.754	79.2	50.636	89.133	−2.0437	1.12E-13	Expansin-A2-like
g3636.t1	25.185	21.582	24.613	59.389	95.21	92.471	−1.7394	1.85E-12	Auxin-induced protein
g61385.t1	7.078	8.562	7.853	23.895	39.57	53.815	−2.4703	7.38E-16	Solute carrier family 22 member 15-like
g1753.t1	8.658	7.508	4.59	28.729	28.726	20.986	−1.9143	1.26E-10	Cytochrome P450 CYP749A22-like
g5301.t1	1.786	1.249	0.395	6.492	7.392	7.745	−2.4252	1.66E-08	Hyoscyamine 6-dioxygenase
g29758.t1	67.734	74.31	62.81	12.817	26.008	26.537	1.70243	2.77E-08	Sucrose transport protein-like
g21304.t1	24.216	25.277	24.157	123.414	147.201	164.917	−2.345	2.44E-37	Bark storage protein A-like

### Gene ontology analysis of DEGs in sweet potato seedlings in response to K^+^ deficiency

**Fig. 4:**
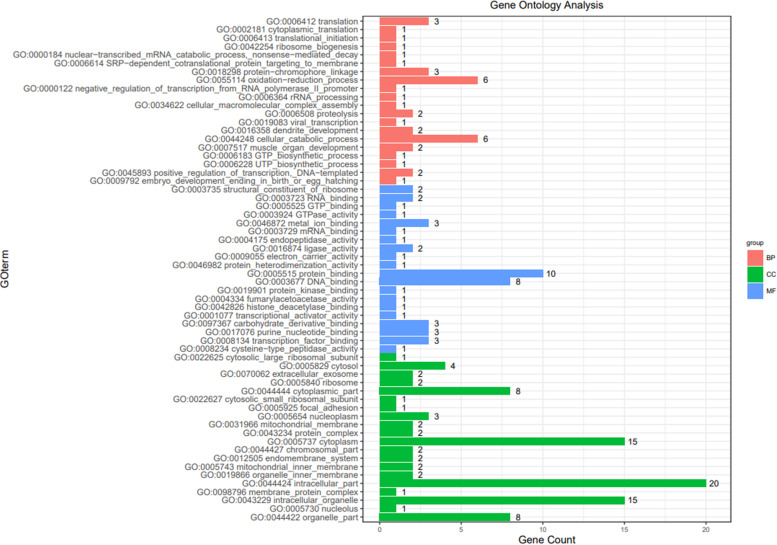
The numbers of DEGs in each GO term was significantly enriched. Functional categorization of genes based on the biological process of gene ontology. Different color indicates an enriched GO category, including BP (biological process); CC (cellular component); MF (molecular function). Each component shows the top 20 of GO term. The x-axis shows the counts of differently expressed genes, and the y-axis shows GO term of gene enriched in each biological process

To evaluate the potential functions of these DEGs in response to K^+^ deficiency, gene ontology (GO) analysis was performed by mapping each DEG into the records of the GO database with an adjusted *p*-value < 0.05 as significant enrichment. We chose the top 20 GO terms for further analysis of biological, cellular component and molecular function processes (Fig. 4). In the GO classification of biological processes, cellular catabolic processes and oxidation-reduction process were significantly enriched, indicating that the ‘Taizhong6’ variety has a wide range of catabolic and oxidation-reduction activities under low-K^+^ stress. Within the ‘cellular component’ classification, intracellular part, cytoplasm, intracellular organelle, and organelle part were prominently represented. Within the ‘molecular function’ classification, the main functional group of DEGs were those related to metal ion binding, protein binding, DNA binding, and nucleic acid binding of TFs (Additional file 3). Most of these processes were closely related to a response to K^+^ deficiency.

### KEGG analysis of DEGs in sweet potato seedlings in response to K^+^ deficiency

**Fig. 5:**
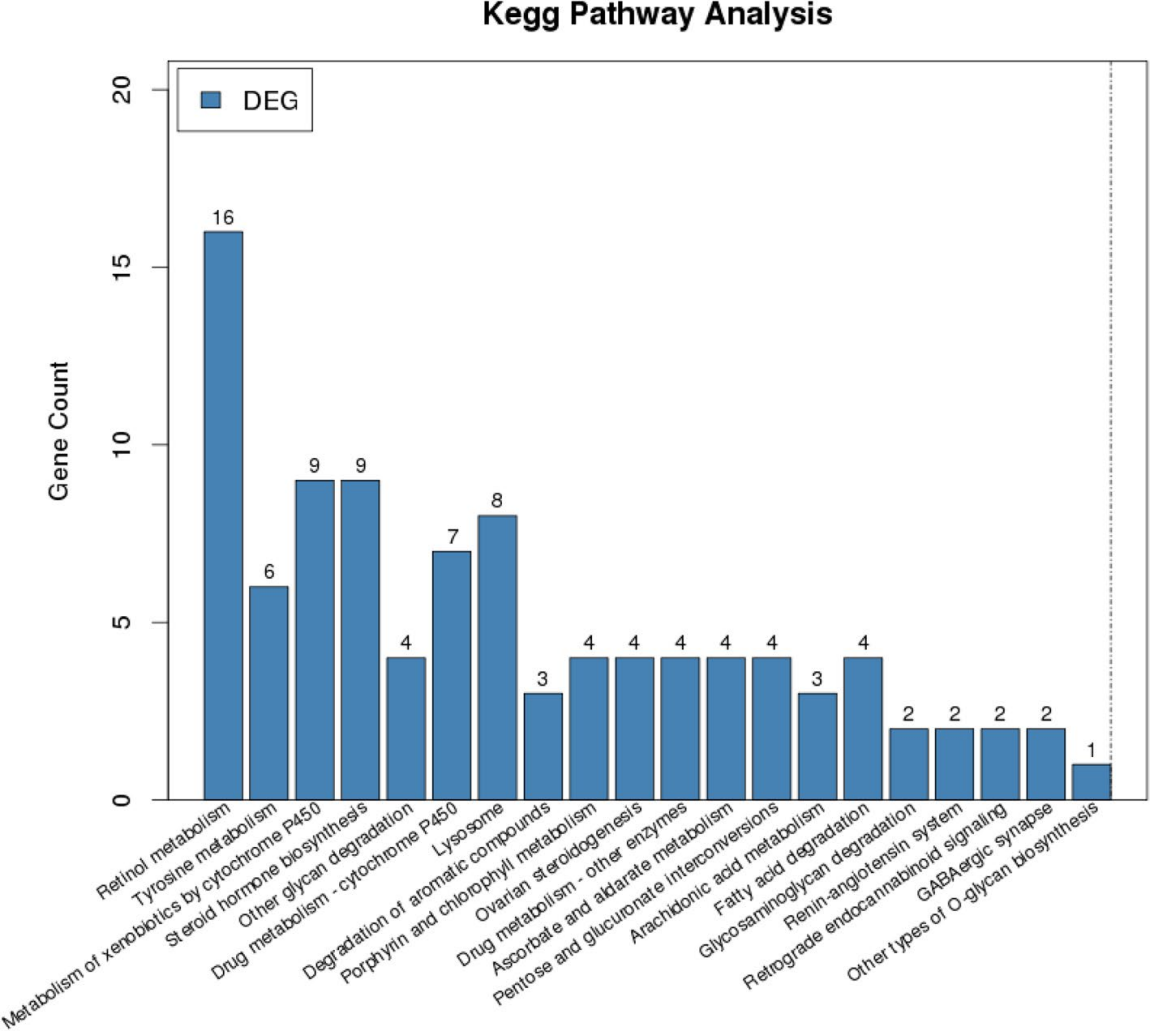
The significantly enriched KEGG pathway of DEGs in sweet potato seedlings root. KEGG pathway analysis shows the top 20 significantly enriched pathway. The x-axis shows different KEGG pathways, and the y-axis shows the numbers of differently expressed genes enriched in each pathway

To identify the pathways in which the DEGs were likely to be involved, we performed the pathway analysis with the KEGG. The top 20 pathways are shown in Fig. 5. The analysis showed that the retinol metabolism, tyrosine metabolism, metabolism of xenobiotics by cytochrome P450, steroid hormone biosynthesis, and other glycan degradations were significantly enriched (Table S5). Previous studies reported that low-K^+^ stress affects the color of fruits and the quality of crops [26, 28, 31]. The cultivar ‘Taizhong6’ used in this study is a carotenoid-rich sweet potato variety and, usually, this orange-fleshed sweet potato is rich in vitamin A. In the transcriptome data from the current study, the retinol (vitamin A) metabolism pathway was the most significant pathway in the KEGG pathway under K^+^-deficient condition (Fig. 5 and Additional file 4). Carotenoids are important source for vitamin A. In this study, the total carotenoids were measured (Fig. S1d), the result showed that there were no significantly difference under normal and K^+^-deficient conditions. The vitamin A of root tuber will have obviously phenotype when K^+^-deficient time is extended. These annotations provide a valuable resource for investigating the response mechanism of sweet potato under low K^+^.

## Discussion

K is an essential macronutrient for physiological processes in plants, and the K supply has a significant effect on crop yield through the regulation of photosynthesis rate and assimilate transport [3]. However, the K^+^ content in each plant is significantly different. Some plants need to take up more K^+^ for their normal life activities, whereas others need little K^+^, suggesting that the mechanisms of K^+^ uptake and assimilation are different. K^+^-enriched plants function especially well when soil K^+^ availability is low and fluctuating, and the plant cells are able to maintain a relative high K^+^ concentration [49].

Low-K^+^ stress affects the growth and development of sweet potato, which requires K to promote storage root formation and bulking [50–53]. The production of sweet potato is remarkably promoted when provided with sufficient K [30]. Genome-wide gene expression profiles have been performed in rice [54, 55], wheat [56], pear [57, 58], cotton [59], and tomato [60]. However, little progress has been made in understanding the transcriptional changes and the K^+^ uptake and utilization mechanism in sweet potato under low-K^+^ conditions. In this study, sweet potato seedling phenotypes under low K^+^ were investigated (Fig. 1a) and genome-wide gene expression profiles were determined under low-K^+^ stress.

Ca^2+^, ethylene and reactive oxygen species (ROS) signature are involved in the response to nutrient deficiency and regulate K^+^ absorption and plant growth. A Ser/Thr protein kinase (CIPK23), which is activated by calcineurin B-like proteins CBL1/9, phosphorylates the K^+^ transporters AKT1 and HAK5 for K^+^ acquisition under low-K^+^ conditions, as mechanism that is found in many crops [9, 47, 61]. In this study, the Ca^2+^ signal related genes are involed in low-K^+^ stress, including genes coding for the calcium/calmodulin-dependent protein kinase and calmodulin-binding transcription activator were both upregulated (Additional file 2). ROS have important roles in many signal transduction pathways by regulating transporter activity [62–64]. Many studies demonstrate that ROS and ethylene accumulated in a discrete region of roots when the plants were deprived of K^+^ [65, 66]. A previous report showed that ethylene acts upstream of the ROS response to potassium deprivation by changing root hair and primary root growth in Arabidopsis [67]. A recent study revealed a mechanism whereby plants sense K^+^ deprivation and how this translates into spatially defined ROS signals to govern specific downstream responses. K^+^ deprivation triggered rapid K^+^ and Ca^2+^ signals in the root K^+^-sensing niche (KSN), meanwhile CIF peptides were induced by Ca^2+^ signals to activate SGN3-LKS4/SGN1 receptor complexes. LKS4 phosphorylated and activated RBOHC/D/F for ROS signal formation, and ROS signals conveyed *HAK5* induction and accelerated casparian strip formation [68]. The ethylene-responsive AP2/ERF transcription factor RAP2.11 was reported to bind to the *AtHAK5* promoter and positively regulated *AtHAK5* expression to enhance absorption of K^+^ under K^+^-deprived conditions [69]. In the current study, ethylene-responsive transcription factor, such as *g5338.t1* (ethylene-responsive transcription factor 4) and *g38192.t1* (ethylene-responsive transcription factor *ERF26*) were upregulated at least four times under low-K^+^ stress. To verified whether transcription factor IbERF can bind to *IbHAK5* promoter, we conducted Y1H assay. We cloned *IbHAK5* gene and analysed its promoter, and we found there are two ERF binding sites in the promoter, expectively ERE box element and ATTTTAAA-motif (Fig. S3 and S4). Yeast-one-hybrid assays showing that IbERF can bind to the fragments of *IbHAK5* promoter (Fig. S3). It implies that IbERF is an important transcription factor in the conditon of potassium deficiency, and further research is under way.

Auxin and jasmonic acid (JA) are also involved in the K signal transduction pathway [45, 70–72]. The auxin response factor ARF2 was reported to bind to the *AtHAK5* promoter to maintain the low expression level of *AtHAK5* under K^+^-sufficient conditions. When low K signals are perceived by plants, ARF2 is phosphorylated, relieving its repression of *HAK5* transcription [73]. The Arabidopsis transcription factor *MYB77*, which modulates auxin signal transduction is down regulated by K^*+*^ deprivation to modulate lateral root development [74]. MYB77 positively regulates the expression of *HAK5* by binding to the *HAK5* promoter and enhances K^+^ uptake by roots [75]. TRH1 is a member of the AtKT/AtKUP/AtHAK family of K carriers and has been identified as an important part of the auxin transport system, affecting root gravitropic behaviour in Arabidopsis [76, 77]. Another member of the AtKT/AtKUP/AtHAK family, *KUP9*, which is highly expressed in quiescent center cells in the root tips maintains Arabidopsis root meristem activity and root growth by regulating K^+^ and auxin homeostasis in response to low-K^+^ stress [25]. In the current study, the auxin-related DEGs *g3636* (homologous gene of nodulin *MtN21*) and *g30544* (homologous gene of *AtPIN8*) were both downregulated under K^+^-deficient conditions. We hypothesize that these auxin-related DEGs are down regulated to reduce plant growth and adapt to the low-K^+^ conditions. MYC2, an important transcription factor related to the JA-responsive signaling pathway, is involved in multiple stress resistances pathways, such as against drought, salt, and fungal pathogens [78–81]. In the current study, a transcription factor similar to *MYC2* was significantly upregulated, suggesting that the JA-response gene *MYC2* is related to low-K^+^ stress (Table 1).

In addition to hormone-related genes involved in K^+^ deficiency, many other genes were found to be changed at the transcriptional level in sweet potato in response to low K^+^. The cell wall is important in plant defense against pathogens [38], and it is hypothesized that crop demands for K^+^ are closely related to cell wall growth. Cell wall-related genes involved in the response to low-K^+^ stress hanve been reported in Arabidopsis [45]. However, little published work supports this hypothesis. In our analysis, cell wall-related transcripts, such as expansin-A2-like, wall-associated receptor kinase-like, and lignin-forming anionic peroxidase, showed significant changes under low-K^+^ conditions (Table 1). These genes were highly expressed under low-K^+^ stress, indicated that K^+^ is associated with cell wall-related activity. K^+^/NO_3_^−^ are generally absorbed and transported in a coordinated manner. CIPK23 was reported to mediate K^+^ uptake by phosphorylating and activating AtAKT1 [47]. Moreover, CIPK23 is involved in regulating nitrate uptake; thus it might act as a connecting node for K^+^ and NO_3_^−^ during early stages of nutrient perception [1, 82]. The nitrate transporter NPF7.3/NRT1.5 functions as a proton-coupled H^+^/K^+^ antiporter with an essential role in K^+^ translocation from root to shoot, and is involved in the coordination of K^+^/NO_3_^−^ distribution in Arabidopsis [48]. In the current study, a high-affinity nitrate transporter was upregulated under low K^+^ stress (Table 1), suggesting that this gene is involved in K^+^ homeostasis in sweet potato under K^+^-deficiency conditions. K is essential for cell turgor and sugar accumulation, especially in fruits, and low K^+^ stress can promote sugar accumulation in the root [58, 83]. In previous reports, low K^+^ stress was closely related to sugar metabolism and signaling [59, 71, 84]. In cassava, increasing the supply of K^+^ led to increased sucrose in the leaves, which is loaded in the phloem and transported to the roots for storage as starch, enhancing root crop yield [85]. Sugars will be eventually effluxed transporter (SWEETs) mediates the transport of sugars across the plasma membrane or the tonoplast [86]. Here, a sugar transport (Table 1) was downregulated in sweet potato under low K^+^ stress and we hypothesize that it might be involved in sugar metabolism and signaling. However, further research is required to explore the function of these key DEGs under K deficiency in sweet potato and other crops.

## Conclusions

**Fig. 6:**
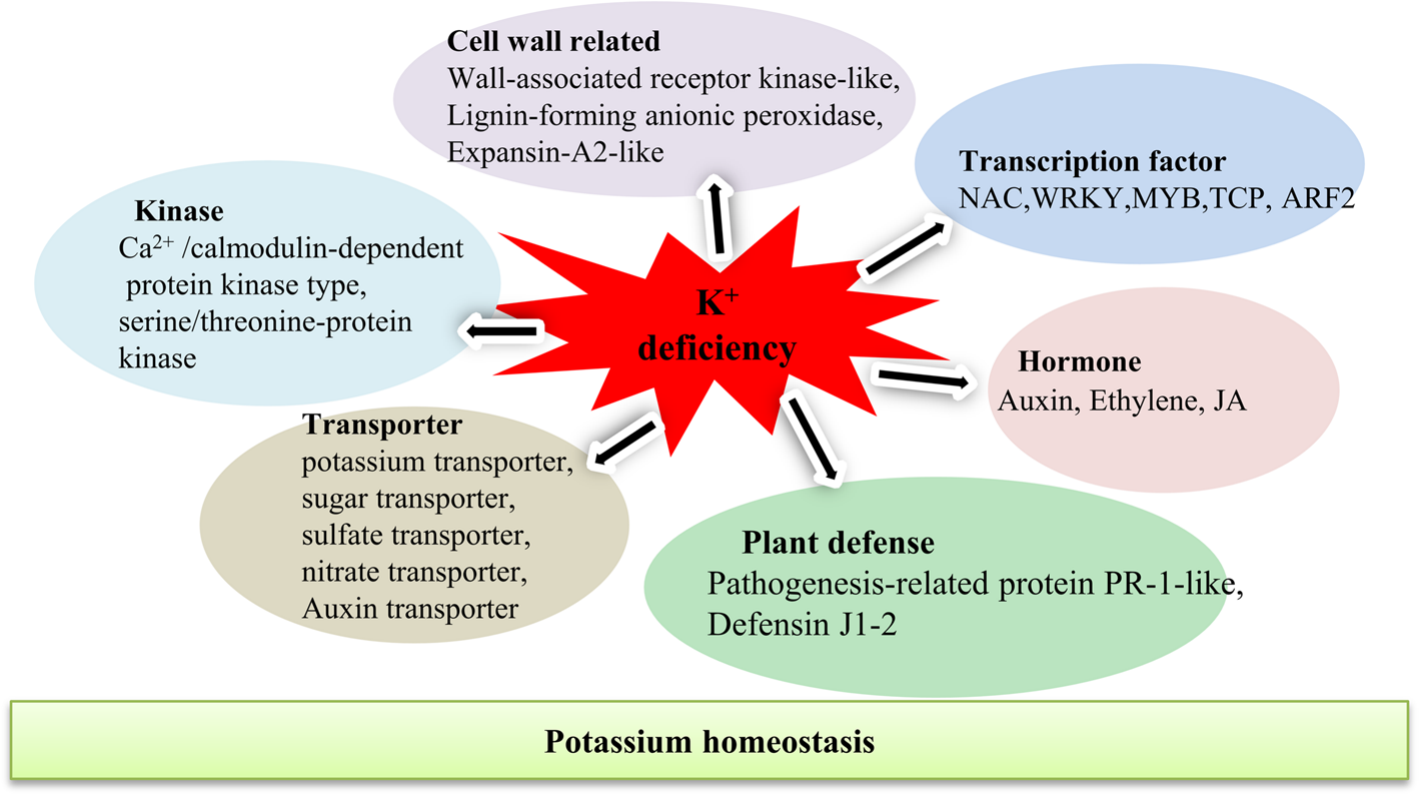
A proposed model of transcription regulation involved in low K^+^ stress in roots. The model is based on the differently expression genes of transcriptome described in this study. When response to potassium deficiency condition, the sweet potato seedlings roots activate multiple methods to maintain the potassium homeostasis

In conclusion, the current study analyzed the transcriptome profiles of sweet potato roots, showing that expression of hundreds of genes changed under low K^+^ stress. Many physiological processes changed in response to low K^+^ stress in sweet potato, and some previously unknow genes were revealed. These genes were associated with the kinase, phytohormone, ROS, Ca^2+^, transcription factors, plant defense, transporters and cell wall (Fig. 6 and Table 1). Those TFs that were significantly altered under low K^+^ condition might positively or negatively regulate K^+^ homeostasis in sweet potato. The network of K^+^ uptake and assimilation in sweet potato is complex, and further research is required to clarify the differences in transcriptional regulation. Thus, this research provides a good reference for the study of the relationship between low-K^+^ stress and other signal pathways in sweet potato and other important crops.

## Methods

### Plant materials and growth condition

‘Taizhong6’, a carotenoid-rich sweet potato cultivar, the China national accession number of which is 2,013,003, was used for this research and its genomic was sequenced [42]. It was propagated by taking 5-cm-long stem cuttings, which were then grown in a plot filled with vermiculite. When the plant reached the two-leaf stage, the seedlings were subjected to either low or high K^+^ under constant illumination with a 13 h light/11 h dark photoperiod, 28 °C day/24 °C night temperatures, and 65% relative humidity for 14 days. Three biological replicates were used for all analyses. All seedlings were watered with modified Hoagland’s solutions every 3 days [87]. The complete nutrient solution (HK, 1 mM K^+^) contained 0.75 mM K_2_SO_4_, 2 mM Ca (NO_3_)_2_.4H_2_O, 0.65 mM MgSO_4_.7H_2_O, 0.1 mM KCl, 0.25 mM KH_2_PO_4_, 0.1 mM Fe-EDTA, 1 mM MnSO_4_, 1 mM ZnSO_4_.7H_2_O, 0.01 mM CuSO_4_.5H_2_O, 0.005 mM (NH_4_)_6_Mo_7_O_24_.4H_2_O, and 1 mM H_3_BO_3_, pH 6.0. For the low-K^+^ groups (0 mM K^+^), KCl and KH_2_PO_4_ were replaced with 0.1 mM NaCl and 0.25 mM NaH_2_PO_4_.2H_2_O; the remaining components were not altered.

### Content measurements

To determine K^+^ content determination, root and shoot tissues were harvested separately from low K^+^ stress plants after 14 days of growth and dried at 80 °C for 72 h. The total K was determined through an atomic absorption spectrophotometer [59]. Three biological replicates were used for the phenotypic test and K^+^ content determination. Student’s *t*-test was used to determine the statistical significance (*P < 0.05, **P < 0.01).

To determine the chlorophyll content, sweet potato stems were cut into 5-cm fragments and grown in plot full with vermiculite to the two-leaf stage. Seedlings were then treated with HK (1 mM K) and LK (0 mM K) Hoagland solution for 14 days, and the shoots were then collected. Chlorophyll was extracted in 80% acetone (v/v) in the dark for 2 days. The absorbance of the extraction buffer at 645 and 663 nm was then measured using microplate reader (BioTek Power Wave XS2) [48]. Three biological replicates were used for this experiment.

The roots of sweet potato seedlings with or without K^+^ treatment were harvested and used for carotenoids determine. The roots were ground with liquid nitrogen and into powder, and 0.1 g power was mixed with 10 mL extraction solvent. The extract set aside in the dark until the residues became colorless. The absorbance of the extract was measured at 440 nm for carotenoid by UV-Visible spectrophotometry [88].

### Genome assembly and transcriptome sequencing

mRNA was isolated from total RNA using magnetic beads with oligo (dT) and fragmentation buffer was added to cut the mRNA into short fragments. cDNA was synthesized using the short fragments as templates through random hexamer-primers. Further dNTPs, RNase H, DNA polymerase I, and buffer solution were added to synthesize the second cDNA. The cDNA fragments were purified with the QiaQuick PCR Purification kit (Qiagen) and resolved with EB buffer for end reparation and poly (A) addition. The ligation products were selected based on size by agarose gel electrophoresis, PCR amplication, and sequencing were performed using an Illumina NovaSeq6000 (the second generation sequencing platform) to conduct the high-throughput transcriptome analysis of the control and low K^+^ stress-treated root samples. Three biological replicates were performed for each treatment.

### RNA-seq read processing, assembly and transcriptome sequence analysis

After quality control, clean reads were mapped separately to the de novo genome assembly using trinity-v2.4.0. ORFs, SNPs, and SSRs were analyzed for each unigene. Each unigene was annotated with the NR (www.ncbi.nlm.nih.gov/), SWISSPROT (www.ebi.ac.uk/uniprot/) and KOG/COG databases (www.ncbi.nlm.nih.gov/research/cog/); GO enrichment analysis (http://geneontology.org/) and KEGG pathway analysis (https://www.kegg.jp/) were then performed for each annotated unigene [89, 90]. Finally, the expression levels of the unigenes were analyzed to reveal those that showed significant differential expression. The *P*-values obtained were adjusted using the Benjamini and Hochberg method for controlling the false discovery rate (FDR). FDR < 0.05 and log2|FoldChange| > 1 were set as the thresholds for significant differential expression. Thus genes with FDR-adjusted *P*-values < 0.05 were defined as DEGs [43]. Gene counts for each sample were imported into RStudio (www.rstudio.com/) with edgeR (www.bioconductor.org/packages/release/bioc/html/edgeR.html/) for DEGs analysis. Lowly expression genes were removed by filtering.

The gene ID used in this study was referenced by using the ‘Taizhong6’ database (https://ipomoea-genome.org/) [42]. In addition, sequencing data were submitted to the National Center for Biotechnology Information Sequence Read Archive (SRA) under accession number PRJNA760652 (www.ncbi.nlm.nih.gov/sra/PRJNA760652). All the annotation information of DEGs used in this research is provided in Additional files 2 and 5.

### RNA isolation, gene cloning and RT-qPCR analyses

For RNA extraction, the shoot and root of sweet potato seedlings were collected after treatment with low K^+^ and complete nutrient solution for 14 days, immediately frozen in liquid nitrogen and stored at − 80 °C. All frozen samples were ground to a powder in liquid nitrogen and weighed (200 mg) to extract total RNA using an RNA extraction kit (EASYspin Plus Complex Plant RNA Kit). Three biological replicates were performed for each treatment. Quality characterization of RNA samples was determined and confirmed using a NanoDrop 2000 fluorospectrometer and formaldehyde denaturing gel electrophoresis.

The two maker genes, *CIPK23* and *HAK5*, which were reported to respond to low K^+^ stress, were cloned from the ‘Taizhong6’ database. The sequences of 11 high DEGs were selected to conform the RNA-seq data. All the gene IDs used in this study were based on the ‘Taizhong6’ database [42]. The primers for these genes are shown in Table S4.

For RT-qPCR analysis, total RNA was treated with DNase I RNase Free (Takara) to eliminate genomic DNA contamination. Then, 10 ng cDNA and 50 nM of each primer were used for each quantitative PCR reaction, which was performed by using Powerup™ SYBR™ Green Master Mix (Applied Biosystems) on a QuantStudio 6 Flex PCR system machine (Thermo Fisher Scientific) following the manufacturers’ protocols. Thermal treatment was 10 min at 95 °C, followed by 40 cycles of 15 s at 95 °C and 1 min at 60 °C. Amplification was followed by a melt curve analysis. The 2^–ΔΔ^Ct method was used for relative quantification and *IbACTIN* was used as an internal reference for data normalization. Three biological replicates were used for each sample. Relative gene expression values were graphed using SigmaPlot v10.0 (Systat Software, https://systatsoftware.com/).

### Yeast one-hybrid assay

The coding sequence of *IbERF* was constructed into the vector pB42AD*.* AD refers to the empty vector expressing the AD domain alone. *LacZ* was used as a reporter gene, driven by the fragments of *IbHAK5* promoter in yeast. The pB42AD-IbERF and Pb42AD plasmid were co-transformed with the *ProIbHAK5:lacZ* plasmids into *Saccharomyces cerevisiae* strain EGY48 using standard transformation techniques respectively. After culturing on SD agar medium lacking Ura and leu (SD/−Ura/−Leu) at 30°Cfor 2 days. Yeast transformants were transferred onto plates SD agar medium lacking Ura and leu containing X-gal (5-bromo-4-chloro-3-indolyl-b-d-galactopyranoside) for blue colour development.

## Supplementary Information

**Additional file 1: Fig. S1.** Phenotypic and quantitative data of sweet potato under normal and K^+^-deficient conditions. a. Phenotype of the root. b. Fresh weight of the shoot and root. c. The dry weight of the shoot and root. d. The content of carotenoids in root under low-K^+^ stress. e. Chlorophyll content of shoot. Sweet potato stems were cut into 5 cm fragments and growth in plot full with vermiculite to two leaf stage. Seedlings were then treated with HK (1 mM K) and LK (0 mM K) Hoagland solution for 14 days, and then the weight, carotenoids and chlorophyll content were detected. Data are shown as means ± SE (n = 4). Student’s *t* test (*P < 0.05) was used to analyze statistical significance, HK-S: sufficient potassium shoot, HK-R: sufficient potassium root, LK-S: low potassium shoot, LK-R: low potassium root, bar=5 cm. **Fig. S2.** The numbers of different unigenes length and venn diagrams of different annotation database. a. Distribution of the numbers of different length unigenes. b. Venn diagrams of transcriptions in each annotation database. **Fig. S3.** Transcription factor ERF binds to *IbHAK5* promoter. a. Diagram of the *IbHAK5* promoter. The adenine residue of the translational start codon ATG was assigned position +1, and the two ERF binding motif were showed in different color. Relative positions of the two motif were indicated by red and green lines. The scale length is 200 bp. b. Transient expression of the *ProIbHAK5:lacZ* fusion together with IbERF in yeast. AD together with *ProIbHAK5:lacZ* was taken as negative control. Observe the color and then photoes were taken. **Fig. S4.** Amino acid sequence alignment of AtHAK5 and IbHAK5. **Table S1.** Summary of RNA-seq quality information. **Table S2.** Unigenes statistical table. **Table S3.** Statistical table of unigenes annotation. **Table S4.** primers used in this study. **Table S5.** Differently expressed unigenes in KEGG.

**Additional file 2: Supplement Table 6.** The raw expression data and annotation of all DEGs under low-K^+^ strss and high potassium condition.

**Additional file 3: Supplement Table 7.** Biological process of genes based on the gene ontology. **Supplement Table 8.** Cellular component of genes based on the gene ontology. **Supplement Table 9.** Molecular function of genes based on gene ontology.

**Additional file 4: Supplement Table 10.** The top 20 KEGG pathways of DEGs under low-K^+^ strss and high potassium condition.

**Additional file 5.** The sequence of all unigenes.

## Data Availability

All data generated or analyzed during this study are included in this article and additional files. The transcriptome sequence was finished in Berry Genomics Corporation (https://www.berrygenomics.com/). The datasets generated in this study are available from the NCBI SRA database under accession number PRJNA760652 (https://www.ncbi.nlm.nih.gov/sra/?term=PRJNA760652). The datasets supporting the conclusions of this article are included within the article (and its additional files).

## Abbreviations

DEG: Differently expressed gene
RT-PCR: Real-time polymerase chain reaction
KUE: K utilization efficiency
LK: Low potassium
HK: High potassium
SNP: Singel-nucleotide polymorphisms
ORF: Open-reading frame
TFs: Transcription factors
ROS: Reactive oxygen species
JA: Jasmonic acid
FC: Fold change
GO: Gene ontology
KEGG: Kyoto Encyclopedia of Genes and Genomes
FDR: False discovery rate
FPKM: Fragments per kilobase of exon per million fragments mapped
