# Supplementary material for: Transcriptome analysis of sweet potato responses to potassium deficiency
Source: BMC Genomics. 2022 Sep 15;23:655. doi: 10.1186/s12864-022-08870-5 (PMC9479357; doi:10.1186/s12864-022-08870-5)
Supplement: Supplementary file 1 — Additional file 1: Fig. S1. Phenotypic and quantitative data of sweet potato under normal and K+-deficient conditions. a. Phenotype of the root. b. Fresh weight of the shoot and root. c. The dry weight of the shoot and root. d. The content of carotenoids in root under low-K+ stress. e. Chlorophyll content of shoot. Sweet potato stems were cut into 5 cm fragments and growth in plot full with vermiculite to two leaf stage. Seedlings were then treated with HK (1 mM K) and LK (0 mM K) Hoagland solution for 14 days, and then the weight, carotenoids and chlorophyll content were detected. Data are shown as means ± SE (n = 4). Student’s t test (*P < 0.05) was used to analyze statistical significance, HK-S: sufficient potassium shoot, HK-R: sufficient potassium root, LK-S: low potassium shoot, LK-R: low potassium root, bar=5 cm. Fig. S2. The numbers of different unigenes length and venn diagrams of different annotation database. a. Distribution of the numbers of different length unigenes. b. Venn diagrams of transcriptions in each annotation database. Fig. S3. Transcription factor ERF binds to IbHAK5 promoter. a. Diagram of the IbHAK5 promoter. The adenine residue of the translational start codon ATG was assigned position +1, and the two ERF binding motif were showed in different color. Relative positions of the two motif were indicated by red and green lines. The scale length is 200 bp. b. Transient expression of the ProIbHAK5:lacZ fusion together with IbERF in yeast. AD together with ProIbHAK5:lacZ was taken as negative control. Observe the color and then photoes were taken. Fig. S4. Amino acid sequence alignment of AtHAK5 and IbHAK5. Table S1. Summary of RNA-seq quality information. Table S2. Unigenes statistical table. Table S3. Statistical table of unigenes annotation. Table S4. primers used in this study. Table S5. Differently expressed unigenes in KEGG. [file 12864_2022_8870_MOESM1_ESM.pptx]

## Slide 1
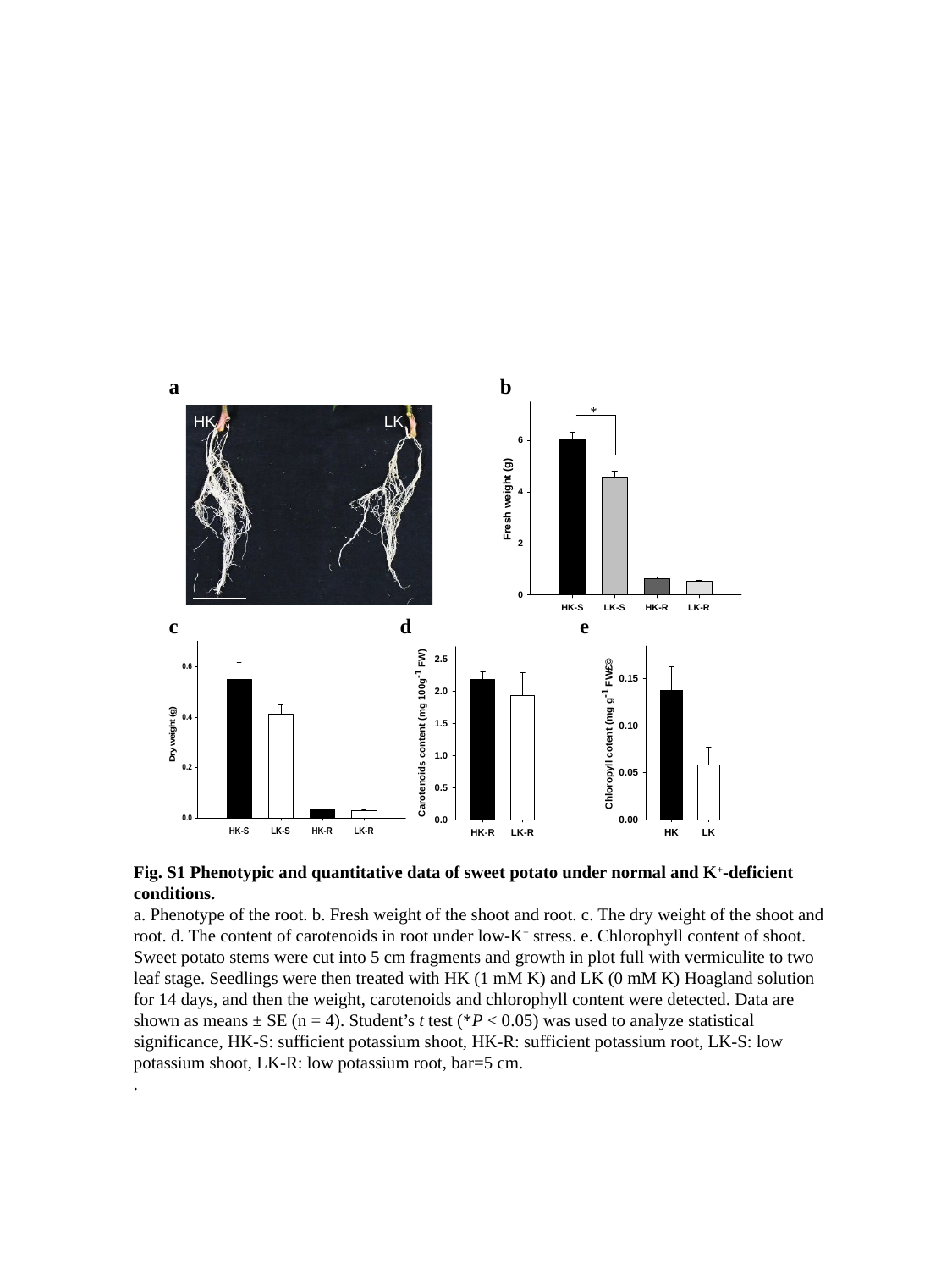

a
b
*
HK LK
c
d
e
Fig. S1 Phenotypic and quantitative data of sweet potato under normal and K+-deficient conditions.
a. Phenotype of the root. b. Fresh weight of the shoot and root. c. The dry weight of the shoot and root. d. The content of carotenoids in root under low-K+ stress. e. Chlorophyll content of shoot. Sweet potato stems were cut into 5 cm fragments and growth in plot full with vermiculite to two leaf stage. Seedlings were then treated with HK (1 mM K) and LK (0 mM K) Hoagland solution for 14 days, and then the weight, carotenoids and chlorophyll content were detected. Data are shown as means ± SE (n = 4). Student’s t test (*P < 0.05) was used to analyze statistical significance, HK-S: sufficient potassium shoot, HK-R: sufficient potassium root, LK-S: low potassium shoot, LK-R: low potassium root, bar=5 cm.
.

## Slide 2
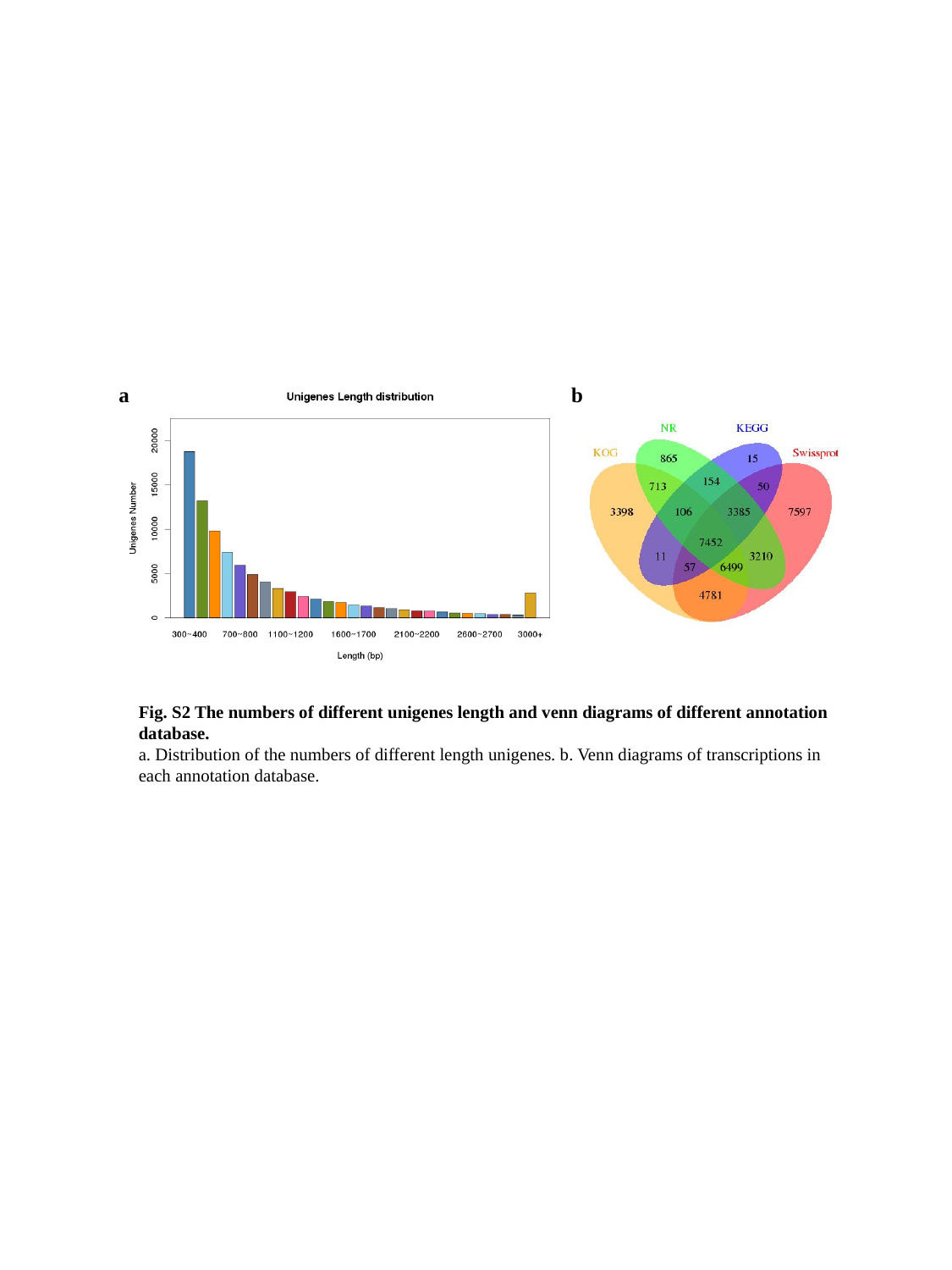

a
b
Fig. S2 The numbers of different unigenes length and venn diagrams of different annotation database.
a. Distribution of the numbers of different length unigenes. b. Venn diagrams of transcriptions in each annotation database.

## Slide 3
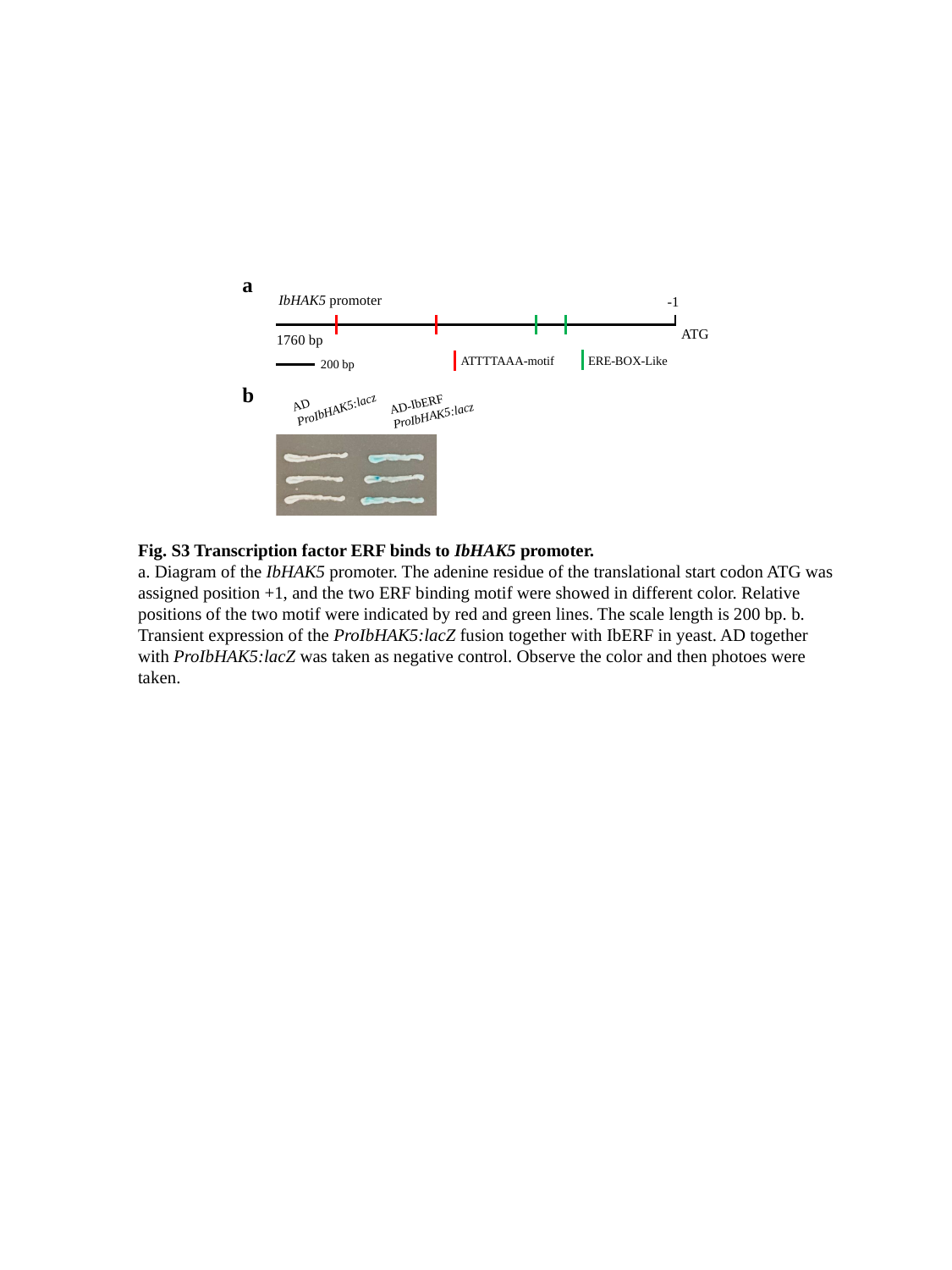

a
IbHAK5 promoter
-1
ATG
1760 bp
ATTTTAAA-motif
ERE-BOX-Like
b
AD
ProIbHAK5:lacz
AD-IbERF
ProIbHAK5:lacz
200 bp
Fig. S3 Transcription factor ERF binds to IbHAK5 promoter.
a. Diagram of the IbHAK5 promoter. The adenine residue of the translational start codon ATG was assigned position +1, and the two ERF binding motif were showed in different color. Relative positions of the two motif were indicated by red and green lines. The scale length is 200 bp. b. Transient expression of the ProIbHAK5:lacZ fusion together with IbERF in yeast. AD together with ProIbHAK5:lacZ was taken as negative control. Observe the color and then photoes were taken.

## Slide 4
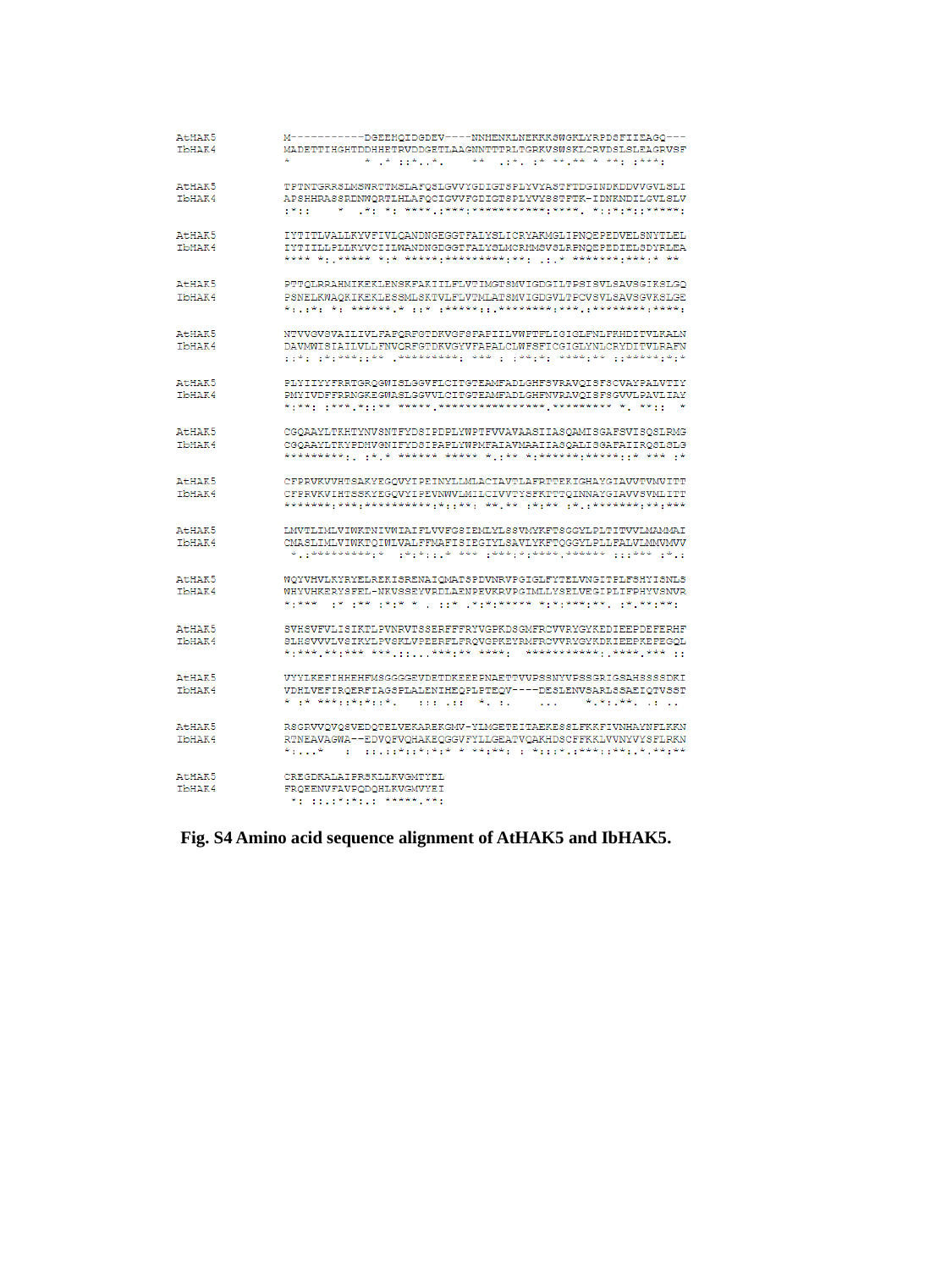

Fig. S4 Amino acid sequence alignment of AtHAK5 and IbHAK5.

## Slide 5
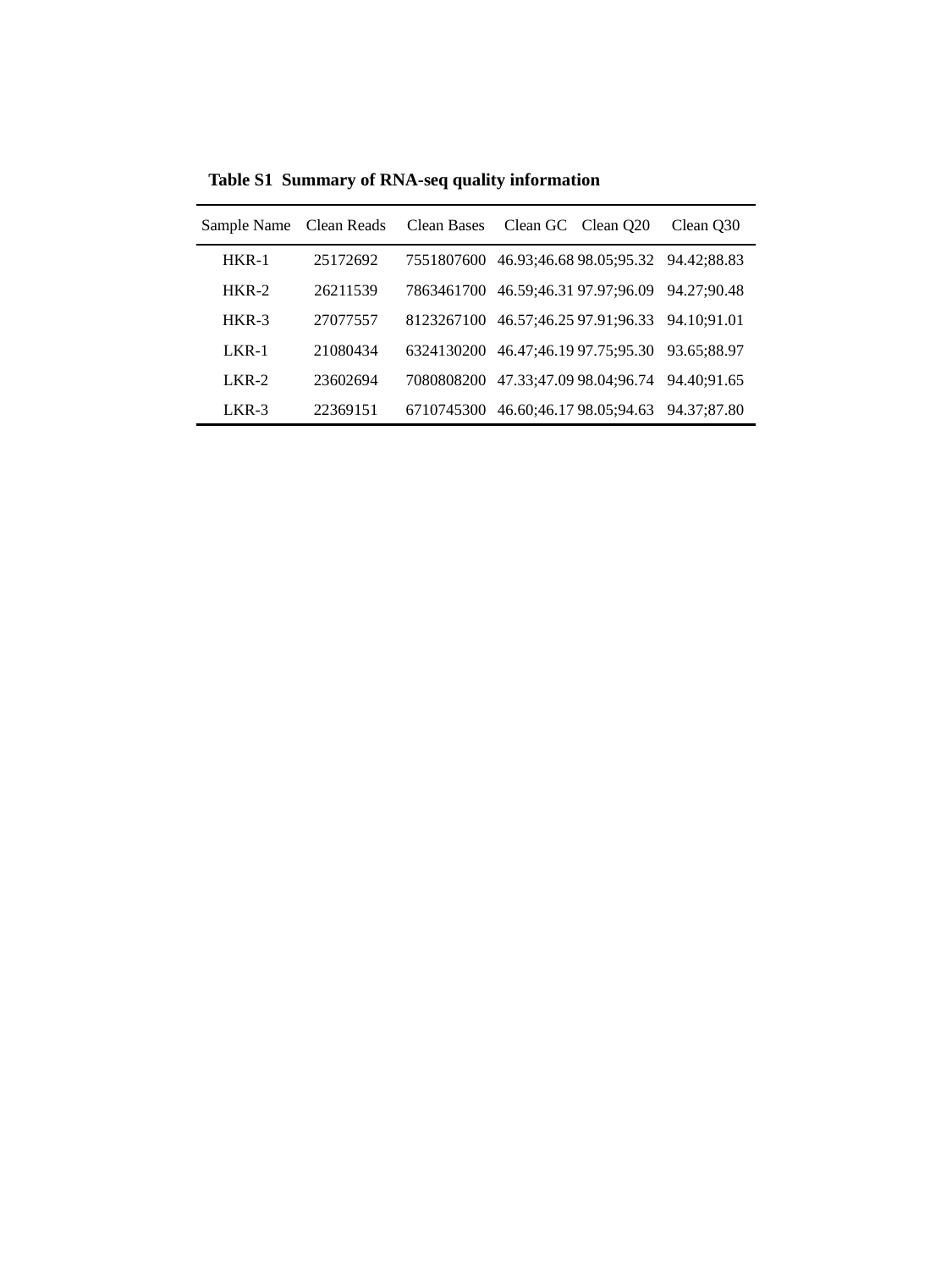

#
Table S1 Summary of RNA-seq quality information
| Sample Name | Clean Reads | Clean Bases | Clean GC | Clean Q20 | Clean Q30 |
| --- | --- | --- | --- | --- | --- |
| HKR-1 | 25172692 | 7551807600 | 46.93;46.68 | 98.05;95.32 | 94.42;88.83 |
| HKR-2 | 26211539 | 7863461700 | 46.59;46.31 | 97.97;96.09 | 94.27;90.48 |
| HKR-3 | 27077557 | 8123267100 | 46.57;46.25 | 97.91;96.33 | 94.10;91.01 |
| LKR-1 | 21080434 | 6324130200 | 46.47;46.19 | 97.75;95.30 | 93.65;88.97 |
| LKR-2 | 23602694 | 7080808200 | 47.33;47.09 | 98.04;96.74 | 94.40;91.65 |
| LKR-3 | 22369151 | 6710745300 | 46.60;46.17 | 98.05;94.63 | 94.37;87.80 |

## Slide 6
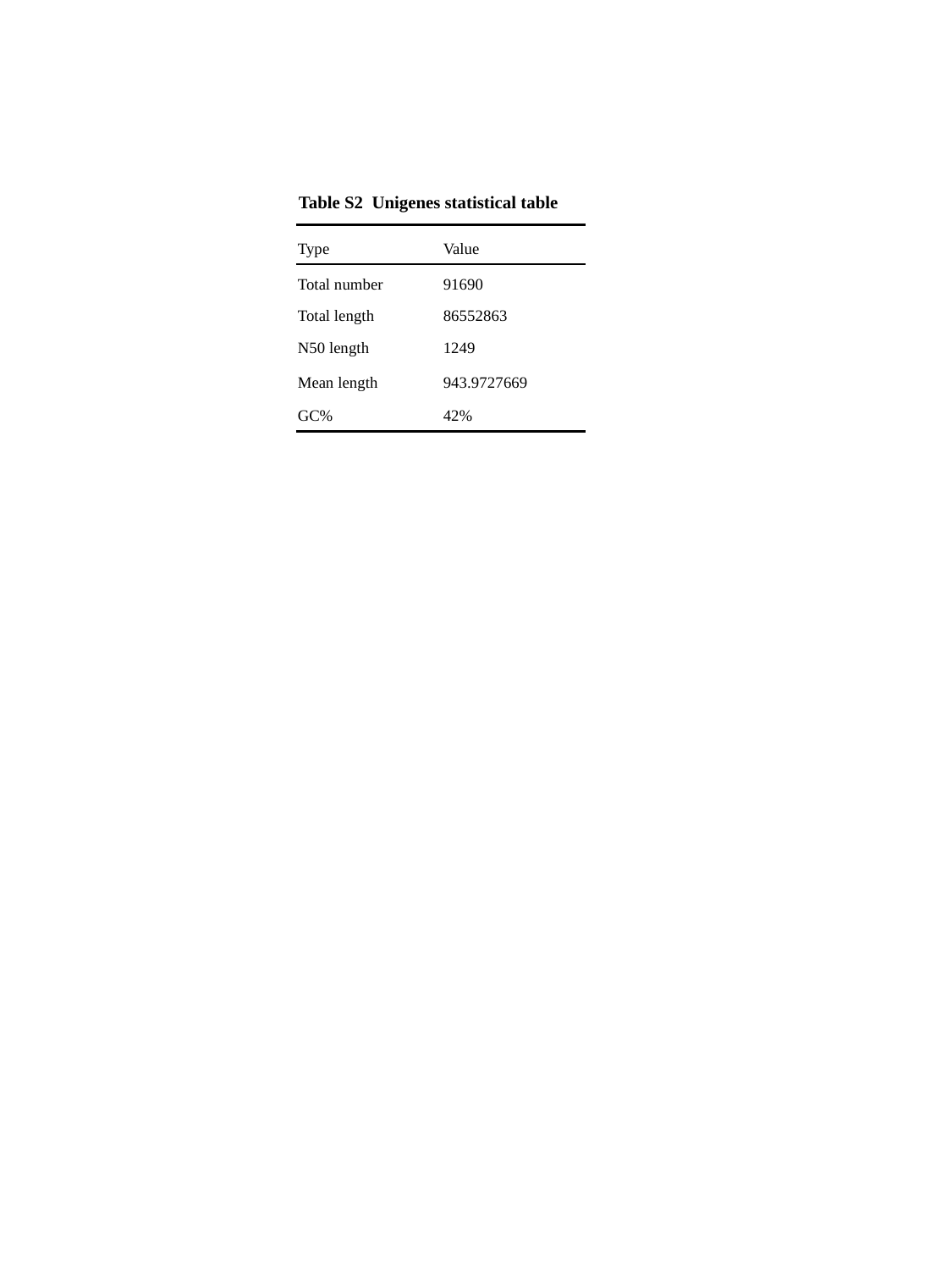

Table S2 Unigenes statistical table
| Type | Value |
| --- | --- |
| Total number | 91690 |
| Total length | 86552863 |
| N50 length | 1249 |
| Mean length | 943.9727669 |
| GC% | 42% |

## Slide 7
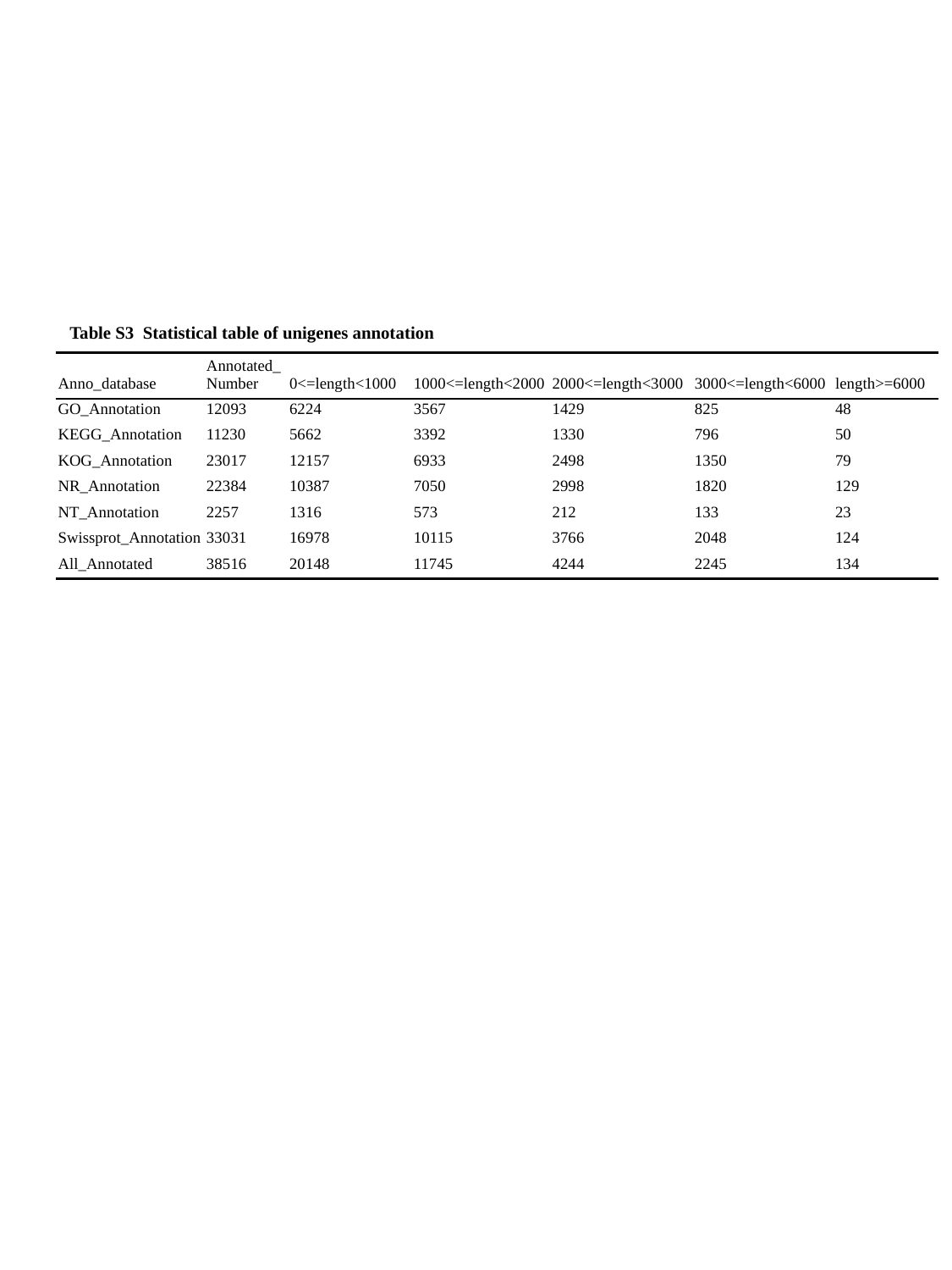

Table S3 Statistical table of unigenes annotation
| Anno\_database | Annotated\_ Number | 0<=length<1000 | 1000<=length<2000 | 2000<=length<3000 | 3000<=length<6000 | length>=6000 |
| --- | --- | --- | --- | --- | --- | --- |
| GO\_Annotation | 12093 | 6224 | 3567 | 1429 | 825 | 48 |
| KEGG\_Annotation | 11230 | 5662 | 3392 | 1330 | 796 | 50 |
| KOG\_Annotation | 23017 | 12157 | 6933 | 2498 | 1350 | 79 |
| NR\_Annotation | 22384 | 10387 | 7050 | 2998 | 1820 | 129 |
| NT\_Annotation | 2257 | 1316 | 573 | 212 | 133 | 23 |
| Swissprot\_Annotation | 33031 | 16978 | 10115 | 3766 | 2048 | 124 |
| All\_Annotated | 38516 | 20148 | 11745 | 4244 | 2245 | 134 |

## Slide 8
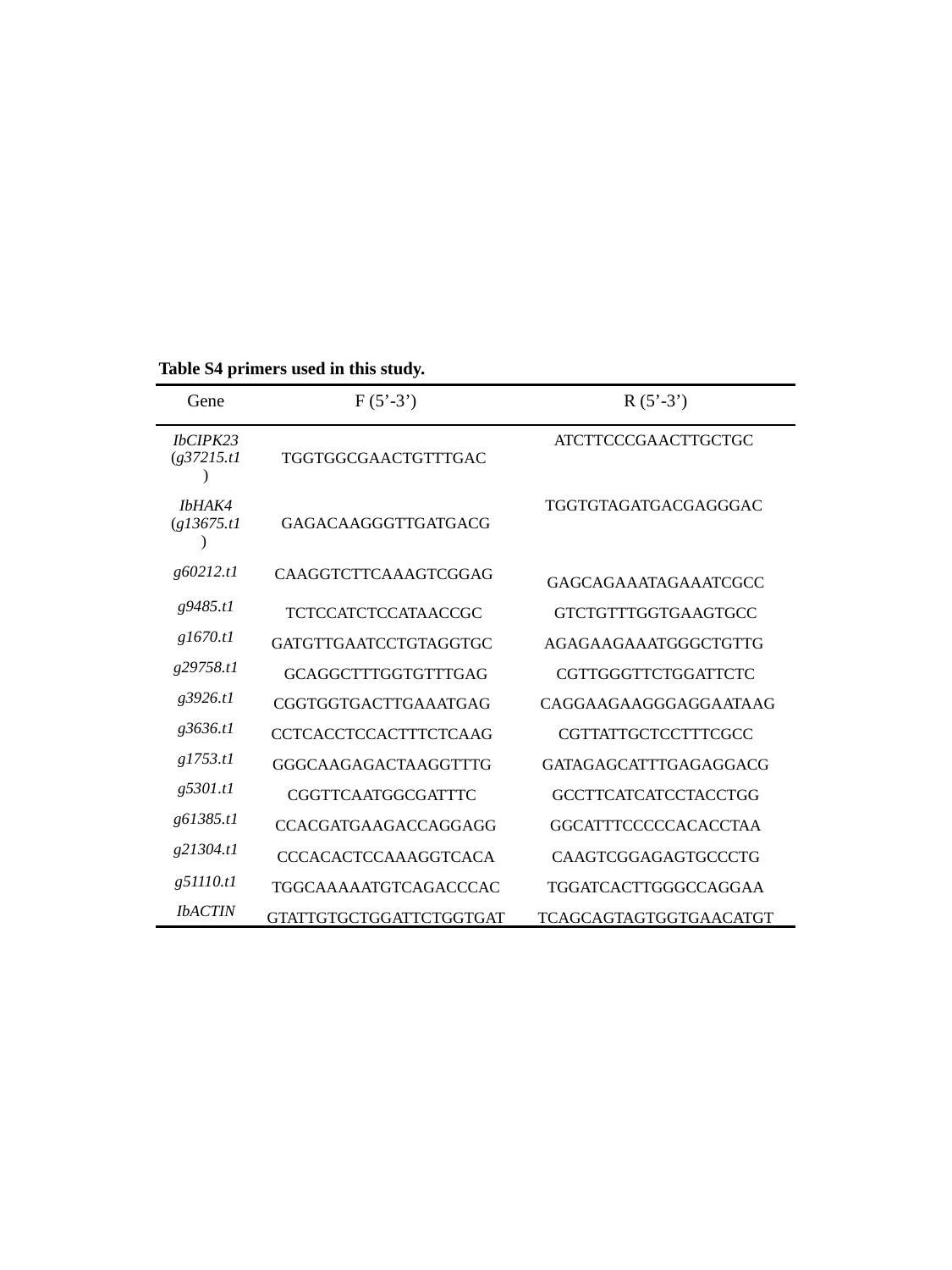

Table S4 primers used in this study.
| Gene | F (5’-3’) | R (5’-3’) |
| --- | --- | --- |
| IbCIPK23 (g37215.t1) | TGGTGGCGAACTGTTTGAC | ATCTTCCCGAACTTGCTGC |
| IbHAK4 (g13675.t1) | GAGACAAGGGTTGATGACG | TGGTGTAGATGACGAGGGAC |
| g60212.t1 | CAAGGTCTTCAAAGTCGGAG | GAGCAGAAATAGAAATCGCC |
| g9485.t1 | TCTCCATCTCCATAACCGC | GTCTGTTTGGTGAAGTGCC |
| g1670.t1 | GATGTTGAATCCTGTAGGTGC | AGAGAAGAAATGGGCTGTTG |
| g29758.t1 | GCAGGCTTTGGTGTTTGAG | CGTTGGGTTCTGGATTCTC |
| g3926.t1 | CGGTGGTGACTTGAAATGAG | CAGGAAGAAGGGAGGAATAAG |
| g3636.t1 | CCTCACCTCCACTTTCTCAAG | CGTTATTGCTCCTTTCGCC |
| g1753.t1 | GGGCAAGAGACTAAGGTTTG | GATAGAGCATTTGAGAGGACG |
| g5301.t1 | CGGTTCAATGGCGATTTC | GCCTTCATCATCCTACCTGG |
| g61385.t1 | CCACGATGAAGACCAGGAGG | GGCATTTCCCCCACACCTAA |
| g21304.t1 | CCCACACTCCAAAGGTCACA | CAAGTCGGAGAGTGCCCTG |
| g51110.t1 | TGGCAAAAATGTCAGACCCAC | TGGATCACTTGGGCCAGGAA |
| IbACTIN | GTATTGTGCTGGATTCTGGTGAT | TCAGCAGTAGTGGTGAACATGT |

## Slide 9
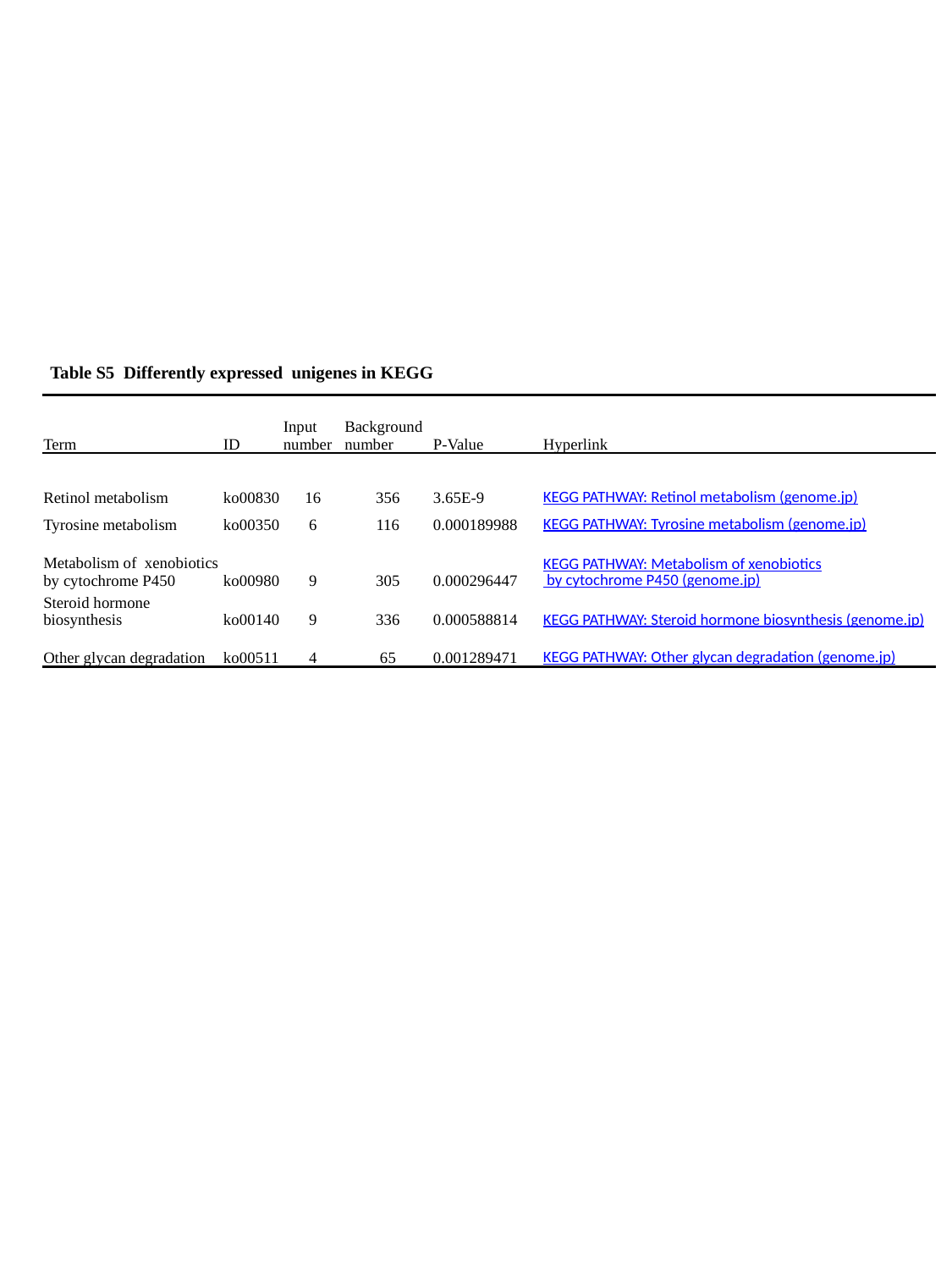

#
Table S5 Differently expressed unigenes in KEGG
| Term | ID | Input number | Background number | P-Value | Hyperlink |
| --- | --- | --- | --- | --- | --- |
| Retinol metabolism | ko00830 | 16 | 356 | 3.65E-9 | KEGG PATHWAY: Retinol metabolism (genome.jp) |
| Tyrosine metabolism | ko00350 | 6 | 116 | 0.000189988 | KEGG PATHWAY: Tyrosine metabolism (genome.jp) |
| Metabolism of xenobiotics by cytochrome P450 | ko00980 | 9 | 305 | 0.000296447 | KEGG PATHWAY: Metabolism of xenobiotics by cytochrome P450 (genome.jp) |
| Steroid hormone biosynthesis | ko00140 | 9 | 336 | 0.000588814 | KEGG PATHWAY: Steroid hormone biosynthesis (genome.jp) |
| Other glycan degradation | ko00511 | 4 | 65 | 0.001289471 | KEGG PATHWAY: Other glycan degradation (genome.jp) |
